# NMDA hypofunction as a convergence point for progression and symptoms of schizophrenia

**DOI:** 10.3389/fncel.2013.00031

**Published:** 2013-03-27

**Authors:** Melissa A. Snyder, Wen-Jun Gao

**Affiliations:** Department of Neurobiology and Anatomy, Drexel University College of MedicinePhiladelphia, PA, USA

**Keywords:** gene, NMDA receptors, psychiatric disorders, neurodevelopment, schizophrenia

## Abstract

Schizophrenia is a disabling mental illness that is now recognized as a neurodevelopmental disorder. It is likely that genetic risk factors interact with environmental perturbations to affect normal brain development and that this altered trajectory results in a combination of positive, negative, and cognitive symptoms. Although the exact pathophysiology of schizophrenia is unknown, the N-methyl-D-aspartate receptor (NMDAR), a major glutamate receptor subtype, has received great attention. Proper expression and regulation of NMDARs in the brain is critical for learning and memory processes as well as cortical plasticity and maturation. Evidence from both animal models and human studies implicates a dysfunction of NMDARs both in disease progression and symptoms of schizophrenia. Furthermore, mutations in many of the known genetic risk factors for schizophrenia suggest that NMDAR hypofunction is a convergence point for schizophrenia. In this review, we discuss how disrupted NMDAR function leads to altered neurodevelopment that may contribute to the progression and development of symptoms for schizophrenia, particularly cognitive deficits. We review the shared signaling pathways among the schizophrenia susceptibility genes DISC1, neuregulin1, and dysbindin, focusing on the AKT/GSK3β pathway, and how their mutations and interactions can lead to NMDAR dysfunction during development. Additionally, we explore what open questions remain and suggest where schizophrenia research needs to move in order to provide mechanistic insight into the cause of NMDAR dysfunction, as well as generate possible new avenues for therapeutic intervention.

## Introduction

Schizophrenia is a devastating psychological disorder that consists of a complex set of positive, negative, and cognitive symptoms. Although the pathophysiological mechanisms associated with this disease remain unclear, the dopamine (DA) hypothesis has dominated the theories of schizophrenia for several decades (Howes and Kapur, 2009; Abi-Dargham, 2012). It was proposed that hyperactivity in the mesolimbic DA pathway is the mediator of positive symptoms of schizophrenia, whereas hypoactivity in the mesocortical DA pathway mediates the negative and cognitive symptoms of schizophrenia. However, focusing on the DA system has led to limited progress in understanding the pathophysiological processes in schizophrenia, and subsequently has led to minimal development of novel therapeutics (Miyamoto et al., 2012). In the past two decades, hypotheses of schizophrenia have progressed beyond the DA hypothesis. In a major paradigm shift on the etiology of schizophrenia, it has been proposed that numerous genetic and environmental risk factors converge on the N-methyl-D-aspartate receptors (NMDAR)-mediated glutamatergic system and result in NMDAR hypofunction in the limbic system during neurodevelopment.

NMDARs are widely thought to be crucial in synaptic plasticity and circuit formation for pre- and early postnatal stages of brain development, otherwise known as the “critical developmental window.” Numerous studies have indicated that the maturation of brain circuitry is usually coincident with the NMDAR subunit switch (e.g., NR2B-to-NR2A and NR3A-to-NR3B) that occurs at the onset of the critical period of development (Monyer et al., 1994; Sheng et al., 1994; Quinlan et al., 1999; Wang et al., 2008; Roberts et al., 2009; Wang and Gao, 2009; Snyder et al., 2013). The NMDAR subunit shift therefore marks the transition from juvenile to “adult” neural processing (Dumas, 2005; Henson et al., 2010) and the subunit switch makes the NMDARs extremely vulnerable to genetic and environmental risk factors (Spear, 2000). Because NMDARs regulate DA neurons and DA transmission, hypofunction of NMDARs may be responsible for the abnormal DA activity associated with the symptoms of schizophrenia. Indeed, the NMDAR-mediated glutamatergic model provides an alternate approach for conceptualizing the brain abnormalities associated with schizophrenia (Harrison and Weinberger, 2005; Lewis and Moghaddam, 2006; Lisman et al., 2008). Although it remains unclear what changes induce the onset of cognitive dysfunction, NMDAR dysfunction appears to be a convergence point for progression and symptoms of schizophrenia, especially for cognitive deficits. There have been several elegant review articles; some issues on a specific topic, such as neuregulin1, circuit-level glutamatergic hypothesis and metabotropic glutamate receptors, can be found in these references (Moghaddam, 2003; Coyle, 2006; Lisman et al., 2008; Banerjee et al., 2010; Marek et al., 2010; Niswender and Conn, 2010; Geddes et al., 2011; Lin et al., 2012; Millan et al., 2012; Vinson and Conn, 2012). Below we focus on the current literature and explain how the hypothesis of NMDA hypofunction is formulated, why NMDA hypofunction could be a convergence point for the progression and symptoms of schizophrenia, what mechanisms are associated with regulation of NMDAR function, as well as possible signaling pathways related to the regulation of NMDAR function by high-risk genes for schizophrenia. It is likely that convergent mechanisms target NMDAR, which in turn contribute to negative symptoms and neurocognitive dysfunction directly (Lau and Zukin, 2007), as well as to positive symptoms via dysregulation of brain DA systems indirectly (Howes and Kapur, 2009; Abi-Dargham, 2012).

## Evidence for abnormal glutamate transmission and NMDAR hypofunction in schizophrenia

In the past two decades, the abnormalities found in human subjects with schizophrenia and the various animal models for schizophrenia all point to an important contribution of the glutamatergic system to the disease (Moghaddam and Jackson, 2003; Javitt, 2004; Millan, 2005). Accumulating studies have shown that aberrant NMDAR function, namely NMDAR hypofunction, in the limbic brain region, may underlie many aspects of molecular, cellular, and behavioral abnormalities associated with schizophrenia (Mohn et al., 1999; Olney et al., 1999; Tamminga, 1999; Dracheva et al., 2001; Krystal et al., 2002; Moghaddam and Jackson, 2003; Javitt, 2004; Coyle, 2006). First, mice with reduced NMDAR expression display behaviors related to schizophrenia (Mohn et al., 1999). Second, NMDAR antagonists, such as phencyclidine (PCP), dizocilpine (MK-801), and ketamine, produce “schizophrenia like” symptoms in healthy individuals (Javitt and Zukin, 1991; Krystal et al., 1994; Lahti et al., 1995). Compelling evidence has suggested that the NMDAR antagonist PCP and its analog compounds can produce a pattern of metabolic, neurochemical, and behavioral changes that reproduce almost exactly those seen in patients with schizophrenia, with remarkable regional specificity (Morris et al., 2005). This finding has provided considerable insight into the processes that lead to the development of the disease, emphasizing the potential importance of NMDAR hypofunction. Third, a majority of the genes that are associated with an increased risk for schizophrenia can influence the function of NMDARs or related receptor-interacting proteins and signal transduction pathways (Moghaddam, 2003; Harrison and Weinberger, 2005) (see below for detail). Fourth, dysregulated NMDAR subunits are usually seen in postmortem tissue from patients with schizophrenia (Akbarian et al., 1996; Gao et al., 2000; Kristiansen et al., 2007; Geddes et al., 2011; Weickert et al., 2012) and in animal models of NMDAR antagonism (Lisman et al., 2008; Gunduz-Bruce, 2009). Postmortem studies also show changes in glutamate receptor binding, transcription, and subunit protein expression in the prefrontal cortex (Akbarian et al., 1996; Kristiansen et al., 2006; Beneyto and Meador-Woodruff, 2008), thalamus (Ibrahim et al., 2000; Clinton and Meador-Woodruff, 2004; Clinton et al., 2006; Dracheva et al., 2008), and hippocampus (Gao et al., 2000; Beneyto et al., 2007; McCullumsmith et al., 2007) of subjects with schizophrenia (Geddes et al., 2011). These changes include decreased NR1, increased excitatory amino-acid transporter, and altered NMDA receptor-affiliated intracellular proteins such as post synaptic density protein 95 (PSD95) and synapse associated protein 102 (SAP102) in the prefrontal cortex and thalamus [see (Geddes et al., 2011) Table 1 for detail]. Fifth, glutamatergic neurons also interact with other neurons that have been strongly implicated in the pathophysiology of schizophrenia, including morphologically altered GABAergic interneurons (Lewis et al., 2005) and antipsychotic drug-targeted DA neurons (Howes and Kapur, 2009; Abi-Dargham, 2012; Grace, 2012).

**Figure 1:**
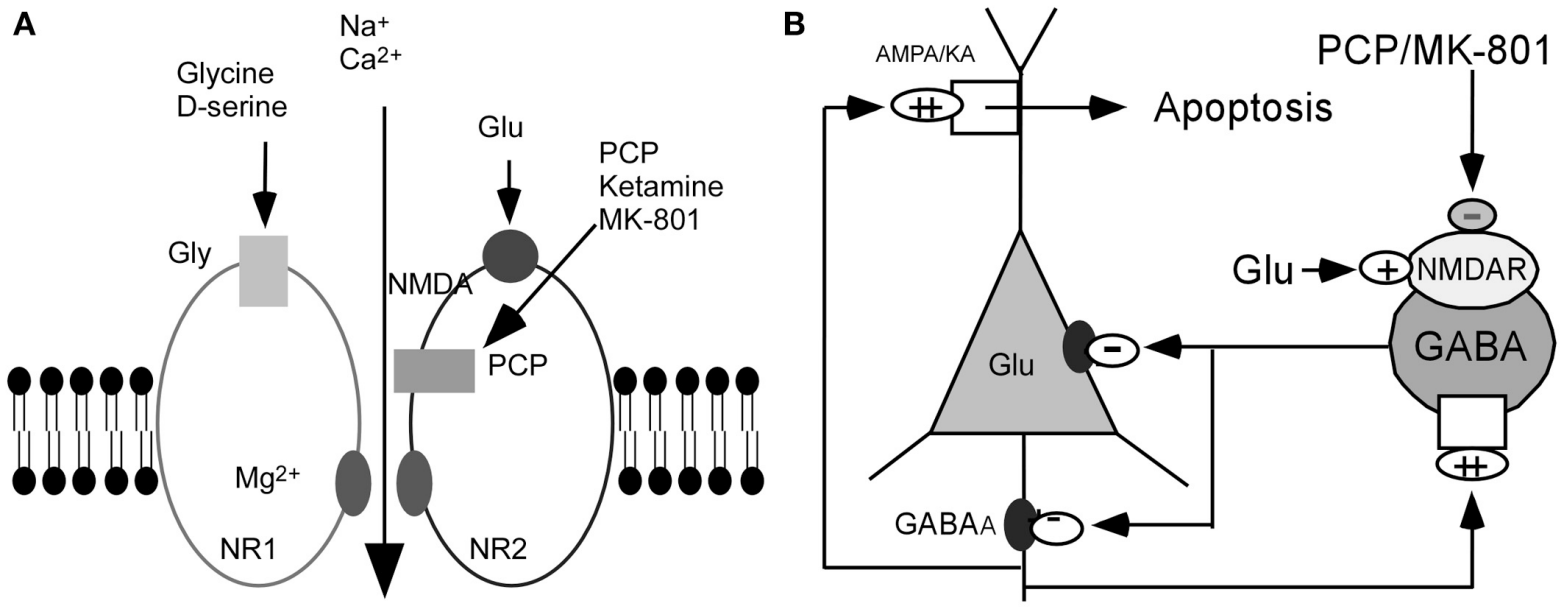
**Hypothesis of NMDAR hypofunction. (A)** Schematic diagram of NMDAR complex. **(B)** NMDAR hypoactivity and glutamate neurotoxicity. PCP/MK801 ⇒ NMDAR hypofunction on GABAergic neurons ⇒ disinhibition of pyramidal neurons ⇒ more glutamate release ⇒ AMPA/KA receptors excessively stimulated ⇒ excitotoxic damage [Figure 1B was modified from (Olney et al., 1999)].

On the basis of these observations, it has been postulated that the glutamatergic disturbances may involve hypofunctioning of NMDARs on gamma-aminobutyric acid (GABA) interneurons in the limbic circuit (Olney and Farber, 1995; Olney et al., 1999; Lindsley et al., 2006; Lisman et al., 2008). How might this be achieved? Activity in the corticolimbothalamic circuit is strongly regulated by local GABAergic interneurons, especially basket and chandelier cells. Output from the cortical pyramidal neurons is suppressed and coordinated by GABAergic interneurons. These cells are activated by recurrent collaterals from the pyramidal neurons and exert a powerful feedback inhibitory action on pyramidal cells via synapses onto the soma and axon hillock (Figure 1). Both basket and chandelier cells are particularly important for restraining excessive pyramidal neuron activity, the impairment of these cells leads to dramatic disinhibition of the pyramidal neuron efferent activity and elevated uncoordinated firing throughout the corticolimbic circuit. Considering the dysfunction of NMDAR subunits in patients with schizophrenia (Akbarian et al., 1996; Eastwood et al., 1997; Goff and Wine, 1997; Grimwood et al., 1999; Gao et al., 2000; Clinton et al., 2003; Clinton and Meador-Woodruff, 2004; Weickert et al., 2012), it has been speculated that NMDAR subunits distributed on interneurons may be responsible for NMDAR hypofunction (Nakazawa et al., 2012). The central pathological characteristics seem to be caused by NMDAR hypofunction acting on GABAergic interneurons, followed by the disinhibition of glutamatergic transmission and an overstimulation of non-NMDARs on pyramidal neurons (Figure 1) (Olney and Farber, 1995; Olney et al., 1999; Lindsley et al., 2006; Lisman et al., 2008). The postulated existence of disinhibited glutamatergic transmission and the subsequent cascade of excitotoxic events resulting from NMDAR hypofunction, degeneration of GABAergic interneurons, or a combination of both, have suggested diverse experimental therapeutic interventions for schizophrenia, such as facilitation of NMDA receptor-mediated neurotransmission and potentiation of GABAergic inhibition (Coyle and Tsai, 2004; Javitt, 2004). Recently, a heuristic model for the pathophysiology of schizophrenia that attempts to reconcile the neuropathological and neurocognitive features of the disorder has been proposed (Lisman et al., 2008).

When does the hypofunction of NMDAR occur and what are the mechanisms involved? Specifically, it is crucial to understand which neurons express altered glutamate receptor subtypes, whether these neurons are inhibitory or excitatory, and how the circuitries are affected. It is possible that the hypofunction of the NMDAR on GABAergic interneurons disrupts the functional integrity of the corticolimbic circuit, causing cognitive impairments and negative symptoms. Based on this hypothesis, it is reasonable to speculate that the NMDARs on frontal cortical and limbic GABAergic interneurons are most sensitive to these antagonists and therefore may be an important site of pathology resulting in NMDAR dysfunction. To address these possibilities, we have examined the developmental changes and functions of NMDARs in identified prefrontal neurons. Interestingly, we found that the development of NR2 subunits in pyramidal neurons and GABAergic interneurons of rat prefrontal cortex is cell type-specific (Wang et al., 2008; Wang and Gao, 2009). NR2B levels remain high until adulthood, without significant NR2B-to-NR2A subunit switch, in layer 5 pyramidal neurons in the prefrontal cortex (Wang et al., 2008); however, they are gradually replaced by NR2A subunits in fast-spiking interneurons (Wang and Gao, 2009). Particularly, fast-spiking interneurons in the prefrontal cortex undergo dramatic changes in glutamatergic receptors during the adolescent period (Wang and Gao, 2009, 2010) and consequently, a cell type-specific change of NMDAR subunits in parvalbumin-positive interneurons is clearly evidenced (Xi et al., 2009). These findings strongly suggested that fast-spiking or parvalbumin-positive interneurons are more sensitive to pharmacological or environmental stimulation. Indeed, we found that MK-801 induces distinct changes of AMPA and NMDARs in the fast-spiking interneurons and pyramidal cells in adolescent rat prefrontal cortex (Wang and Gao, 2012). Furthermore, when the NR1 subunit was selectively eliminated in parvalbumin-positive interneurons in forebrain cortices and hippocampus in early (neonatal) development, the rats exhibited reduced glutamic acid decarboxylase 67 (GAD67) and parvalbumin as well as distinct schizophrenia-related symptoms that emerged after adolescence; in contrast, post-adolescent deletion of NR1 did not result in such abnormalities (Belforte et al., 2010). These basic studies in NMDAR development in the prefrontal cortex have been extremely useful in the formulation of an NMDAR hypofunction hypothesis. The high vulnerability of corticolimbic fast-spiking interneurons to genetic predispositions and early environmental insults such as excitotoxicity and oxidative stress could help to better explain their significant contribution to the development of schizophrenia (Nakazawa et al., 2012). Given that both DA and GABA systems are indeed the targets of NMDAR disruption, it is plausible to propose that dysfunction of NMDARs in the DA neurons and GABAergic cells induce DA hyperactivity or GABA downregulation, which in turn results in psychosis.

Still, this does not completely explain the pathophysiology of schizophrenia, as there is evidence of NMDAR dysfunction in other key brain areas, especially during development. In addition to the prefrontal cortex, the hippocampus is a brain region that is consistently implicated in schizophrenia (Bogerts et al., 1990; Medoff et al., 2001; Harrison, 2004; Witthaus et al., 2009). In hippocampus, like other cortical regions, proper NMDAR subunit expression and function is necessary for hippocampal development, with NMDAR misregulation affecting synaptogenesis and circuit maturation (Roberts et al., 2009; Brigman et al., 2010; Gambrill and Barria, 2011; John Gray et al., 2011). Therefore, misregulation of NMDAR subunit composition and function during hippocampal development may contribute to the pathogenesis in schizophrenia. Indeed, we recently found in the MAM neurodevelopmental schizophrenia model, that NMDAR function is disrupted in CA1 pyramidal neurons early in hippocampal development (Snyder et al., 2013). Understanding when and how NMDAR function is disrupted in regards to schizophrenia progression is a key area of research.

## Schizophrenia is a neurodevelopmental disorder with multiple susceptibility genes converging on NMDARs

It is increasingly recognized that schizophrenia is a neurodevelopmental disorder that involves disrupted alterations in brain circuits (Weinberger, 1987; Lewis and Gonzalez-Burgos, 2008; Jaaro-Peled et al., 2009). Although psychosis usually emerges in late adolescence or early adulthood, we still do not understand all of the changes in normal or abnormal development prior to and during this period. It is particularly unclear what factors alter the excitatory-inhibitory synaptic balance in the juvenile brain and what changes induce the onset of cognitive dysfunction. Current studies suggest that problems related to schizophrenia are evident much earlier than the juvenile stage of development. The emerging picture from genetic and epigenetic studies indicates that early brain development is affected. However, after many years of intensive investigations, no single gene has been found to be responsible for schizophrenia. Although recent findings have generated great interest in the copy number variations of genes in schizophrenia patients, they are rare and are unlikely to account for the majority of cases of the disorder (Allen et al., 2008; O'Donovan et al., 2008; Stefansson et al., 2008). Rather, a number of high-risk genes have been identified as increasing susceptibility for schizophrenia (Allen et al., 2008), including the catechol-o-methyltransferase gene (COMT) (Weinberger et al., 2001; Bilder et al., 2004; Cannon, 2005; Harrison and Weinberger, 2005; Savitz et al., 2006; Tunbridge et al., 2006; Tan et al., 2009), neuregulin 1 (NRG1) (Roy et al., 2007; Mei and Xiong, 2008; Kato et al., 2011), disrupted in schizophrenia-1 (DISC-1) (Lipina et al., 2010; Niwa et al., 2010), and dystrobrevin-binding protein 1 (dysbindin) (Iizuka et al., 2007; Ji et al., 2009; Papaleo and Weinberger, 2011; Papaleo et al., 2012), among others. Many of these genetic variants associated with schizophrenia are involved with neurodevelopment that is related to the glutamatergic system in the brain (Hahn et al., 2006; Allen et al., 2008; Shi et al., 2008; Papaleo et al., 2012).

Recent studies indicate that single genes may not be sufficient to cause schizophrenia. Instead, multiple “susceptibility” genes could possibly work together to trigger disease onset with each susceptibility gene coding for a subtle molecular abnormality in transmitter receptors, enzymes, protein kinases, transcription, and translation (Harrison and Weinberger, 2005). These subtle changes could disrupt neurodevelopment, intracellular signaling pathways and neurotransmission, consequently resulting in disturbed information processing in brain circuits that mediate the symptoms of schizophrenia. It is therefore not surprising that many of the susceptibility genes for schizophrenia regulate not only neuronal proliferation, neuronal migration, and synaptogenesis during early development, but also have functions linked to glutamate neurotransmission, especially the NMDA receptor, in postnatal development (Straub and Weinberger, 2006; Karam et al., 2010).

Numerous susceptibility genes have been shown to be able to regulate various elements of NMDAR mediated signaling. Dysbindin, neuregulin, and DISC1 all function to affect NMDAR function through a variety of mechanisms. Both dysbindin and neuregulin regulate the formation and function of the postsynaptic density (PSD), a set of proteins that interacts with the postsynaptic membrane to provide structural and functional regulatory elements for neurotransmission and for NMDARs (Numakawa et al., 2004; Hahn et al., 2006). Neuregulin also activates an Erb signaling system that is co-localized with NMDARs (Hahn et al., 2006). This Erb signaling system is a member of the receptor tyrosine kinase and neurotrophin signal transduction system, interacts with PSD, and is involved in neuroplasticity mediated by NMDARs (Huang et al., 2000). Furthermore, neuregulin has been shown to alter NMDAR expression (Ozaki et al., 1997; Li et al., 2007; Mei and Xiong, 2008; Banerjee et al., 2010) [see (Geddes et al., 2011) for detail]. Preventing NRG1/ErbB4 signaling leads to loss of NMDA synaptic currents and dendritic spines (Li et al., 2007). Dysbindin also regulates the activity of the vesicular glutamate transporter, vGluT (Fanous et al., 2005), and may contribute to NMDAR dysfunction (Karlsgodt et al., 2011). Furthermore, the degree of dysbindin-induced NR1 degradation correlates with impairment in spatial working memory performance (Karlsgodt et al., 2011). This is strong evidence that dysbindin's effects on NMDAR expression could contribute to the cognitive symptoms of schizophrenia.

DISC1 affects presynaptic glutamate release from axonal terminals (Maher and LoTurco, 2012), and regulates cyclic adenosine monophosphate (cAMP) signaling, which would affect the functions of glutamate neurotransmission mediated by metabotropic glutamate receptors (mGluR) (Millar et al., 2005). DISC1 also binds to and stabilizes serine racemase (SR), the enzyme that generates D-serine, an endogenous co-agonist of the NMDA receptor. In a mouse model of selective and inducible expression of mutant DISC1 in astrocytes, the main source of D-serine in the brain, Ma et al. found that mutant DISC1 leads to SR degradation, resulting in D-serine deficiency that coincides with behavioral changes indicative of altered NMDAR neurotransmission (Ma et al., 2012). While not yet specifically tested, these changes would likely lead to reduced function of NMDARs at synapses. In addition, the DAOA gene encodes a protein that activates the enzyme D-amino acid oxidase, which degrades the co-transmitter D-serine that acts at glutamate synapses and at NMDARs. DAOA activates this enzyme, so abnormalities in this gene would be expected to alter the metabolism of D-serine, which in turn would alter glutamate neurotransmission at NMDARs (Stahl, 2007a).

Thus, there is strong evidence that the known susceptibility genes for schizophrenia converge on glutamate synapses, specifically at NMDARs. These observations support the notion that the NMDAR hypofunction hypothesis is a plausible theory for schizophrenia (Stahl, 2007a) and NMDAR dysfunction is a convergence point for schizophrenia (Kantrowitz and Javitt, 2010). Genes that code for any subtle molecular abnormalities linked to NMDAR function in specific brain circuits theoretically could create inefficient information processing at glutamate synapses that can produce the symptoms of schizophrenia, especially cognitive dysfunctions. If these genetically mediated abnormalities occur simultaneously in a permissive environment, the syndrome of schizophrenia could be induced and onset of symptoms will be triggered (Stahl, 2007b).

## Molecular mechanisms associated with NMDAR regulation and NMDAR hypofunction in schizophrenia

As discussed above, there are many risk genes associated with schizophrenia. However, changes in their expression and function are unlikely to entirely account for the pathophysiology of schizophrenia. A fundamental question is what causes the alteration of NMDAR during neurodevelopment in schizophrenia. In addition to genetic modifications, there are several possible mechanisms, including altered transcription/translation and posttranslational modifications that could contribute to NMDAR hypofunction in schizophrenia. For example, NMDAR hypofunction could result from reduced levels of mRNA and translation and in fact, there is evidence of reduced mRNA levels of some NMDAR subunits in postmortem tissue of schizophrenics (Dracheva et al., 2001; Beneyto and Meador-Woodruff, 2008; Weickert et al., 2012) but plenty of evidence also suggests an increase or no change in some subunits (Akbarian et al., 1996; Geddes et al., 2011; Weickert et al., 2012). Given the complexity of the disorder and the numerous risk genes involved, it is likely that several mechanisms work in concert. Fortunately, substantial knowledge exists as to how NMDARs are translated, trafficked to synaptic membranes, stabilized, exocytosed, and removed for recycling or degradation (Sans et al., 2003; Wenthold et al., 2003; Perez-Otano and Ehlers, 2004; Lau and Zukin, 2007). However, any disruption of this well-regulated process can lead to NMDAR hypofunction and contribute to altered development and symptomatology seen in schizophrenia. Thus, it becomes a daunting challenge to understand the pathophysiological processes involved.

An exciting avenue of research in schizophrenia and other psychiatric disorders is evaluating the epigenetic changes that occur in these illnesses. Epigenetics is a broad term that describes changes to chromatin which alter the frequency of gene transcription without changing the genetic sequence. These changes include DNA methylation and a variety of histone modifications. In general, increasing DNA methylation, particularly at CpG islands of promoter sequences, will decrease gene expression (Bird, 2002). Therefore, even if a gene is not found to be definitively altered in human schizophrenic patients by standard genome-wide association study (GWAS), it is possible that epigenetic changes are contributing to altered neurodevelopment and cognitive symptoms in schizophrenia (Borrelli et al., 2008; Day and Sweatt, 2011; Rodenas-Ruano et al., 2012). Indeed, a role for histone acetylation and methylation in cognition is increasingly being appreciated (Jeremy Day and Sweatt, 2011). Other data suggest that chromatin modifications by histone deacetylases (HDACs) may underlie cognitive dysfunctions in a variety of mental disorders (Fischer et al., 2010). Thus far, epigenetic modulation of several genes, including GAD1 and RELN, has been found to be altered in schizophrenia (Abdolmaleky et al., 2005; Ruzicka et al., 2007). Additionally, the DNA methylating enzyme, DNA-methyltransferase 1 (DNMT1), showed increased expression in cortical interneurons in postmortem tissue from schizophrenics (Veldic et al., 2005). This change in DNMT1 correlated with the alterations in GAD1 and RELN. However, it is possible that other genes and associated interacting proteins are also similarly affected. For example, animal research has shown that NMDAR subunit expression can be altered through various epigenetic changes (Stadler et al., 2005; Jiang et al., 2010; Rodenas-Ruano et al., 2012). Furthermore, DNA methylation changes have been found in the promoter sequence for NR3B in major psychosis (Mill et al., 2008). These studies suggest that epigenetic regulation of NMDARs could contribute to the pathophysiology of schizophrenia. Still, it is unclear how epigenetic factors control the expression of NMDARs, particularly mRNA expression of individual subunits. It is possible that CpG islands in the promoter region of a NMDAR subunit are regulated by chromatin modification (Rodenas-Ruano et al., 2012). Gene mutation or environmental risk factors could alter gene promoter sequences via either DNA methylation or histone modification and thus result in mis-expression of NMDARs.

Furthermore, NMDAR subunits undergo several post-translation modifications including phosphorylation, palmitoylation, and polyubiquitination. Dysregulation of any of these processes can greatly impact channel function and expression and consequently contribute to NMDAR hypofunction. The most-studied posttranslational modification of NMDARs is phosphorylation, which is a well-characterized means for regulating synaptic localization, stabilization, and channel kinetics. Therefore, changes in NMDAR phosphorylation have important implications both for synaptic plasticity and cognitive symptoms in schizophrenia (Rosenblum et al., 1996; Lu et al., 1998; Li et al., 2009). This dynamic process not only involves the direct phosphorylation of NMDARs, but also kinase activation and subsequent phosphorylation of other synaptic proteins (Lau and Zukin, 2007; Lau et al., 2010). Moreover, the NR2 subunit's large C-terminus has many putative sites for phosphorylation which can affect channel gating and stabilization at the synapse (Monyer et al., 1992; Kornau et al., 1995). NMDAR subunits are phosphorylated at serine or threonine and at tyrosine residues (Raymond et al., 1994; Wang and Salter, 1994; Kohr and Seeburg, 1996; Tingley et al., 1997). These sites are substrates for phosphorylation by a variety of kinases including the Src family of kinases (SFK), cAMP-dependent protein kinase A (PKA), protein kinase C (PKC), cyclin-dependent kinase 5 (Cdk5), casein kinase 2 (CK2), and CaMKII (Omkumar et al., 1996; Raman et al., 1996; Li et al., 2001; Chung et al., 2004). In fact, the activity and expression of many of these kinases are altered in postmortem tissue from human schizophrenic patients (Aksenova et al., 1991; Engmann et al., 2011; Funk et al., 2012). This provides strong evidence that altered kinase signaling likely plays a role in NMDAR function in schizophrenia.

It is clear that the interaction between synaptic scaffolding proteins and the NR2 subunit C-terminal tails are critical for NMDAR synaptic targeting and thus could contribute to NMDAR hypofunction. PDZ-containing proteins can bind directly to NR2 subunits via PDZ recognition sequences in the distal portions of their C-termini, and this association is critical for targeting NDMARs to the synapse (Mori et al., 1998; Steigerwald et al., 2000; Lin et al., 2004). Further, both NR2A and NR2B are known to interact with membrane-associated guanylate kinase (MAGUK) family of proteins, including PSD-95, PSD-93, and SAP102 (Al-Hallaq et al., 2007). Interestingly, the neuregulin receptor ErbB4 also associates with similar PDZ domains, positioning NRG-Erb signaling to affect NMDAR function (Garcia et al., 2000). Furthermore, ErbB4 interacts with FYN, a member of SFKs. SFKs phosphorylate tyrosine residues on both NR2A and NR2B subunits affecting channel gating and increasing NMDAR currents (Wang and Salter, 1994; Kohr and Seeburg, 1996; Hisatsune et al., 1999; Nakazawa et al., 2001; Takasu et al., 2002). NRG1-Erb signaling can prevent Src upregulation of NMDAR-mediated currents by inhibiting NR2B phosphorylation (Li et al., 2001; Bjarnadottir et al., 2007; Pitcher et al., 2011). Additionally, NMDAR tyrosine phosphorylation is important for synaptic plasticity. NR2B tyrosine phosphorylation is increased following long-term potentiation (LTP) and inhibiting Src activation prevents LTP induction (Grant et al., 1992; Rosenblum et al., 1996; Rostas et al., 1996; Lu et al., 1998). In hippocampus, NRG-Erb signaling can suppress LTP (Kwon et al., 2005; Pitcher et al., 2008). Therefore, NRG1 could contribute to cognitive dysfunction in schizophrenia by altering NMDAR function and/or affecting synaptic plasticity (Mei and Xiong, 2008). Similarly, DISC1 is a known binding partner of PDE4B, which regulates cAMP activity and thus PKA activity (Millar et al., 2005; Clapcote et al., 2007). PKA-mediated phosphorylation of NMDARs can affect their release from the endoplasmic reticulum, and regulate expression levels of NR2B (Scott et al., 2003; Llansola et al., 2004). However, it has not been directly tested whether mutations in DISC1 affect NMDAR expression and function. Additionally, it remains an open question if disruption of dysbindin would produce similar modifications in NMDARs. If and how the schizophrenia risk genes affect NMDAR phosphorylation and thus expression and function is an area of research that needs to be further explored.

Another crucial mechanism for proper NMDAR function is the maintenance of appropriate levels of NMDARs in the synapse. This process requires a balance between NMDAR insertion and endocytosis. Specialized endocytic zones involving clathrin-coated pits have been described lateral to the PSD for glutamatergic synapses, and serve to internalize NMDARs (Blanpied et al., 2002; Petralia et al., 2003; Nong et al., 2004). Altered dysbindin expression can alter NMDAR surface expression through clathrin-dependent endocytosis (Jeans et al., 2011). Further, palmitoylation and ubiquitination can also regulate NMDAR synaptic numbers. Palmitoylation is a reversible process that involves the covalent attachment of palmitate group to proteins via thioester bonds at cysteine residues. Palmitoylation is a critical regulator of many cellular processes involved in neuronal development and synaptic plasticity (Fukata and Fukata, 2010). Therefore, dysregulation of palmitoylation could contribute to synaptic dysfunction and cognitive symptoms in schizophrenia. Furthermore, key proteins implicated in schizophrenia, including GAD65 and PSD-95 are known to be regulated dynamically through palmitoylation (El-Husseini Ael et al., 2002; Kanaani et al., 2008). More recently, it was discovered that palmitoylation can regulate NR2A and NR2B trafficking (Hayashi et al., 2009). In fact, palmitoylation can promote synaptic stabilization or sequestering of NMDARs in the Golgi apparatus to affect the level of NMDARs at synapses. Interestingly, altered protein palmitoylation was found in a mouse model of 22q11.2 deletion, a high risk factor of developing schizophrenia (Madry et al., 2008). However, it remains unknown if NMDAR palmitoylation is disrupted in schizophrenia and if or how other schizophrenia risk genes may be involved.

Equally as important as trafficking and stabilizing proteins in the synapse is the process of targeting proteins for removal and degradation. It is known that ubiquitin-based protein degradation of NMDARs is an important homeostatic regulator of NMDAR levels at synapses (Ehlers, 2003). For example, down-regulation of synaptic NR1 has been associated with polyubiquitination (Groblewski and Stafford, 2010; Bangash et al., 2011). Additionally, ubiquitination of scaffolding proteins, such as Shank3, is linked to NR2B downregulation (Mao et al., 2009a). Also, NR2B itself is ubiquitinated in a Fyn dependent manner (Jurd et al., 2008). Given NRG1-ErbB4 interactions with Fyn, it is possible that their signaling could contribute to ubiquitination of NR2B. However, this relationship has not been tested experimentally. Therefore, while there is evidence that the ubiquitin proteasome pathway is disrupted in schizophrenia (Nilsson et al., 2007), it is currently unknown how ubiquitination of NMDARs and other synaptic proteins contribute to the disease process. Exploring this relationship as well as how schizophrenia risk genes could alter these processes is an important line of research.

Given the diverse set of mechanisms that could contribute to NMDAR hypofunction, it is not surprising that multiple signaling pathways are implicated in schizophrenia. For example, both PLC/IP3R/Ca^2+^ and Ras/MEK/ERK (extracellular signal-regulated kinase) signaling pathways are involved in the neuregulin-induced reduction of NMDAR currents, which likely occurs through enhancing NR1 internalization via an actin-dependent mechanism (Gu et al., 2005). While the candidate genes discussed activate many signaling cascades to affect neurodevelopment and NMDAR function, the AKT (also known as protein kinase B) signaling pathway, and its downstream target glycogen synthase kinase 3β (GSK-3β) may serve as a convergence point or common pathway. AKT is a serine/threonine kinase that serves in a variety of processes including regulation of protein synthesis, neurodevelopment, and neuronal plasticity (Sanna et al., 2002; Jiang et al., 2005; Balu et al., 2012). Further, DISC1, NRG1, and dysbindin all contribute to these cellular processes, and are all known regulators of AKT and GSK3β (Lemke, 1996; Huang et al., 2000; Kamiya et al., 2005; Ghiani et al., 2010; Lee et al., 2011). DISC1 regulates the AKT-GSK3β signaling pathway to affect neurodevelopment and adult neurogenesis (Kim et al., 2009; Mao et al., 2009b). Furthermore, knockdown of DISC1 with siRNA caused a decrease in AKT phosphorylation, which would in turn increase GSK3β activity (Hashimoto et al., 2006). Interestingly, reducing GSK3β activity was able to correct behavioral deficits in DISC1 mutant mice, strongly implicating DISC1 affects GSK3β in schizophrenia pathogenesis (Lipina et al., 2011, 2012). Similarly, both NRG1 and dysbindin can regulate AKT phosphorylation (Numakawa et al., 2004; Guo et al., 2010). Additionally, AKT protein levels and phosphorylation of GSK3β are altered (Emamian et al., 2004) and NRG1-stimulated phosphorylation of AKT is reduced in schizophrenia (Keri et al., 2009). Yet, how would regulation of the AKT/GSK3β signaling pathway by DISC1, NRG1, and dysbindin affect NMDAR function? It was recently demonstrated that GSK3β activity can regulate NMDAR expression and function (Li et al., 2009; Xi et al., 2011). While this evidence provides a possible common link between schizophrenia risk genes and NMDAR hypofunction, direct experimental evidence is still needed.

## Conclusion and future perspective

In this review, we have summarized the current literature and discussed the various mechanisms that are associated with NMDAR regulation in schizophrenia. All of the findings derived from the known genetic risk factors for schizophrenia suggest that NMDARs may serve as a convergence point for the progression and symptoms of schizophrenia. Despite such progress, there are still many questions that need to be answered to confirm this intriguing hypothesis. For example, it is unclear how gene mutations in neurons and/or astrocytes and their interaction can lead to NMDAR dysfunction during development. It is also unknown how disrupted NMDAR function leads to altered neurodevelopment, which contributes to the progression and development of this devastating disease. The vast majority of schizophrenia research has focused on changes in adulthood, leaving neurodevelopmental alterations relatively unexplored. So, while it is known that proper expression and regulation of NMDARs is critical for cortical maturation and synaptic plasticity that underlie cognitive functioning, it is unknown if there is a common signaling pathway, such as AKT/GSK-3β pathway, mediates this pathophysiological process among the schizophrenia susceptibility genes. If yes, what are the downstream target substrates of AKT and/or GSK-3β that contribute to the regulation of NMDAR functions? It is possible that AKT/GSK-3β act directly upon NMDARs as our recent research suggests (Li et al., 2009; Xi et al., 2011). However, given their diverse targets (Kockeritz et al., 2006; Peineau et al., 2008; Karam et al., 2010; Li and Gao, 2011), it is also possible they indirectly affect NMDARs by acting on other targets, such as β-catenin (Mao et al., 2009b), β-arrestin (Beaulieu et al., 2005), DISC1 and/or PDE4 interaction (Mao et al., 2009b; Lipina et al., 2012), as well as the AKT/mTOR signaling pathway. Activation of mTOR has been functionally linked with local protein synthesis in synapses, resulting in the production of proteins required for synaptic formation and maturation (Kelleher et al., 2004; Hoeffer and Klann, 2010).

In addition, although psychosis manifests primarily in late adolescence or early adulthood, the emerging picture from genetic and epigenetic studies indicates that early brain development is affected, and cognitive symptoms, such as learning and memory deficits, are evident much earlier. Specifically, schizophrenia may progress from risk to prodrome in the early stage until onset of psychosis and chronic disability in the late stage (Insel, 2010). Therefore, theoretically, the key to forestall the disorder is to detect and prevent early stages of risk and prodrome with novel therapeutic targets for early treatment (Lieberman et al., 2006; Insel, 2010). However, in general, schizophrenia-related research has focused on how NMDAR function in adults contributes to psychosis and cognitive symptoms. These findings, although intriguing, are limited in that they do not reveal the changes before psychosis, specifically during neurodevelopment. In fact, there is no consensus among animal models to what changes occur pre-pubertally and how the genetic susceptibilities interact. Does the process occur simultaneously or sequentially, with various changes culminating in altered development? If it is a sequential process, when do these changes occur and is there a point of no return in terms of preventing cognitive symptoms and psychosis? It appears that adolescence is a critical period for onset of psychosis, but how and by what mechanisms? Therefore, in studying molecular mechanisms that underlie the pathophysiology of schizophrenia and related disorders, a sharp focus on the specific neurodevelopmental trajectory, especially in early development and adolescent brain maturation, is vitally important (Jaaro-Peled et al., 2009; Insel, 2010). Animal studies, particularly developmental models, will certainly help to reveal the neurodevelopmental trajectory of schizophrenia, yield disease mechanisms, and eventually offer opportunities for the development of new treatments, especially for early treatment of cognitive deficits. Utilizing multiple animal models to address similar questions will provide the greatest opportunity for determining consistent changes that most likely contribute to the progression of schizophrenia. It would also be important to definitively determine which neurons express altered glutamate receptor subtypes, whether these neurons are inhibitory or excitatory, and how the circuitries are affected by these high-risk genes.

Furthermore, it is critical to determine if there comes a point during neurodevelopment where brain circuitry is sufficiently altered that no therapeutics will halt the progression of the disease. At present, there are no approved medications for the treatment of either negative symptoms or cognitive dysfunction in schizophrenia (Ibrahim and Tamminga, 2011). However, new pharmacological and behavioral approaches aimed at potentiating glutamatergic neurotransmission, particularly at NMDARs, offer new hope for future clinical development. Although many studies support the theory of NMDAR hypofunction, they do not address the very important conceptual question of whether early treatment with mGluR agonists or other agents is able to prevent the progression or reverse the cognitive deficits and even psychosis that occur in the late stage of the disease. A failure to correct mutant phenotypes with treatment administered after symptom onset would suggest a missed critical period window and indicate that schizophrenia is a terminally differentiated phenotype of altered brain development. Earlier theories supported the notion that effective treatment for developmental disorders such as schizophrenia and autism could only occur during the critical developmental window, after which the brain would be hard wired. Indeed, recent studies demonstrated that a comprehensive phenotype correction is possible with pharmacological intervention (mGluR5 inhibitor) starting in young (3–5 postnatal weeks) animals, after development of the phenotype, in both a Fragile X syndrome model (Michalon et al., 2012) and Shank-2 knockout mice (Won et al., 2012). In addition, adolescent administration of mGluR5 PAMs not only reverse adult-onset deficits, but also prevent the emergence of cognitive impairment induced by neonatal treatment with PCP in a developmental model of schizophrenia (Clifton et al., 2013). These findings certainly offer fresh hope for schizophrenia treatment, suggesting that NMDARs could be critical targets for treatment. Currently, our experiments are under way to test this hypothesis in rats with gestational methylazoxymethanol exposure (Snyder et al., 2013) and other animal models.

Finally, if NMDAR dysfunction in schizophrenia is relative, rather than absolute, enhanced practice might be able to overcome reduced plasticity. Given the number of convergent mechanisms that may contribute to impaired NMDAR function, ideal treatment in schizophrenia may engage optimizing function within a number of convergent pathways. Combinatorial pharmacological and behavioral interventions, rather than simply targeting the point of convergence, may prove to be the most successful strategy in treating schizophrenia symptoms. Nevertheless, focusing on NMDAR hypofunction provides a wonderful opportunity for correcting cognitive impairment in schizophrenia disease progression.

### Conflict of interest statement

The authors declare that the research was conducted in the absence of any commercial or financial relationships that could be construed as a potential conflict of interest.

## Abbreviations

Akt: also known as Protein Kinase B (PKB), is a serine/threonine-specific protein kinase
AMPAR: α-amino-3-hydroxy-5-methyl-4-isoxazole propionic acid receptor
BDNF: brain derived neurotrophic factor
cAMP: cyclic adenosine monophosphate
CaMKII: Ca2+/calmodulin dependent protein kinase II
cdk5: cyclin-dependent kinase 5
CK2: casein kinase 2
COMT: catechol-o-methyltransferase
DA: dopamine
Dysbindin: also known as dystrobrevin-binding protein 1
DISC1: disrupted in schizophrenia-1
DAOA: D-amino acid oxidase activator
HDAC: histone deacetylase
DNMT1: DNA-methyltransferase 1
ERK: extracellular-signal-regulated kinase
GABA: gamma-aminobutyric acid
GAD65: glutamic acid decarboxylase 65
GAD67: glutamic acid decarboxylase 67
GSK-3β: glycogen synthase kinase 3β
LTP: long-term potentiation
MAGUK: membrane-associated guanylate kinase
mGluR: metabotropic glutamate receptor
MK801: dizocilpine
NMDAR: N-methyl-D-aspartate receptor
NRG1: neuregulin 1
PCP: phencyclidine
PKA: protein kinase A
PKC: protein kinase C
PLC: phospholipase C
PSD95: post synaptic density protein 95
SAP102: synapse associated protein 102
SFK: Src family of kinases
PDE4B: cAMP-specific phosphodiesterase 4B
SR: serine racemase
vGluT: vesicular glutamate transporter.
